# Supplementary material for: Metabolic multireactor: Practical considerations for using simple oxygen sensing optodes for high-throughput batch reactor metabolism experiments
Source: PLoS One. 2023 Jul 11;18(7):e0284256. doi: 10.1371/journal.pone.0284256 (PMC10335663; doi:10.1371/journal.pone.0284256)
Supplement: S17 File — Temperature was increased and decreased by immersing the water reservoir in a water heater or an ice bath, respectively. (DOCX) [file pone.0284256.s017.docx]

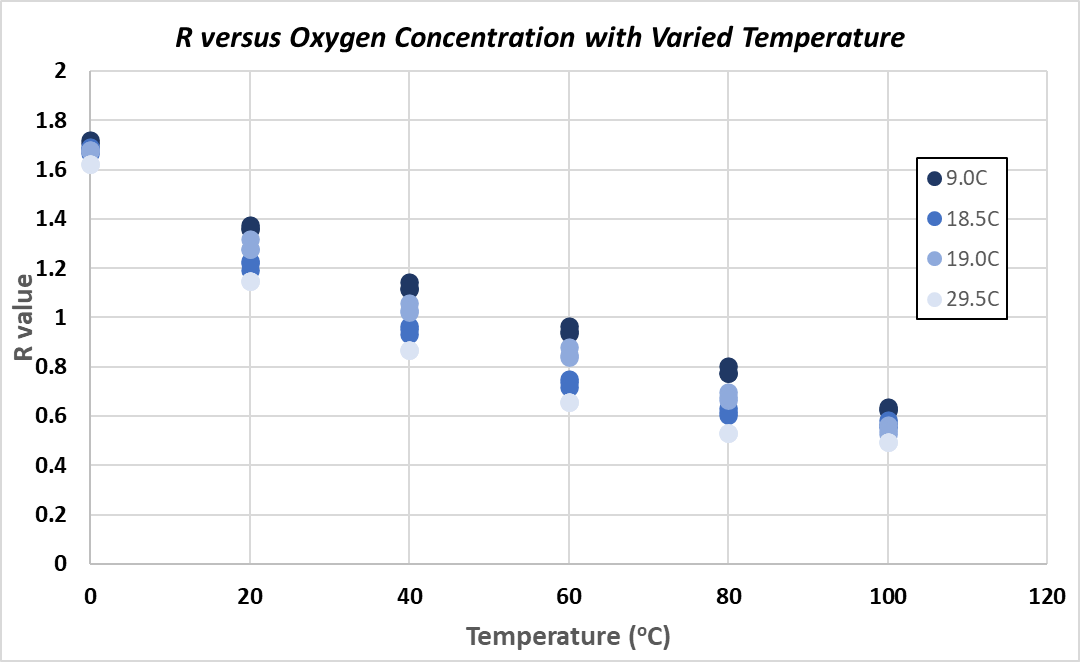


S17: Oxygen saturation vs. R values with temperature varied from 9.0^o^C to 29.5^o^C. Temperature was increased and decreased by immersing the water reservoir in a water heater or an ice bath, respectively.
