# Supplementary material for: Metabolic multireactor: Practical considerations for using simple oxygen sensing optodes for high-throughput batch reactor metabolism experiments
Source: PLoS One. 2023 Jul 11;18(7):e0284256. doi: 10.1371/journal.pone.0284256 (PMC10335663; doi:10.1371/journal.pone.0284256)
Supplement: S18 File — The dashed line approximates room temperature, the upper line indicates measurements made at 9.0°C, and the lower line indicates measurements made at 30°C. The x-axis represents actual oxygen measurement values under ambient temperatures (~19°C) and the y-axis represents the resulting measurements at varied temperature. (DOCX) [file pone.0284256.s018.docx]

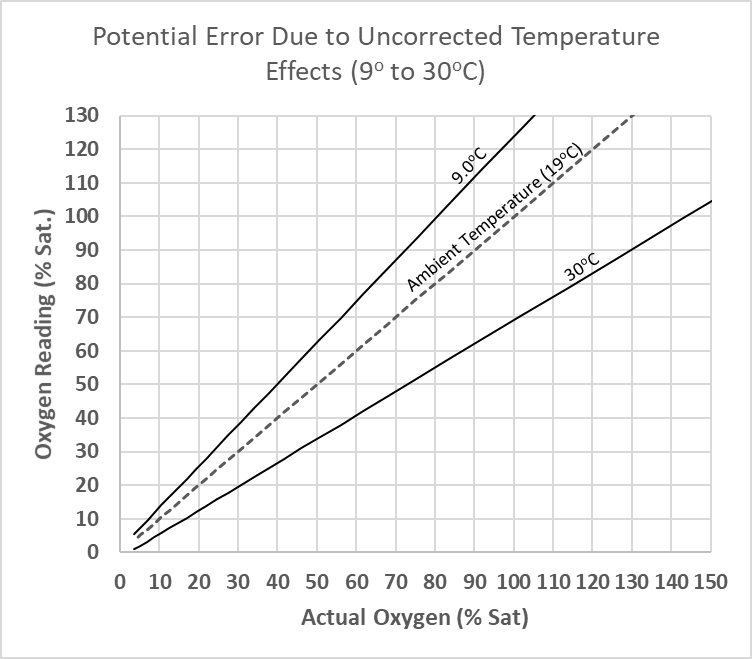


S18: Plot depicting potential error ranges for uncorrected temperature effects (in systems ranging from 9^o^C to 30^o^C. The dashed line approximates room temperature, the upper line indicates measurements made at 9.0^o^C, and the lower line indicates measurements made at 30^o^C. The x-axis represents actual oxygen measurement values under ambient temperatures (~19^o^C) and the y-axis represents the resulting measurements at varied temperature.
