# Supplementary material for: Metabolic multireactor: Practical considerations for using simple oxygen sensing optodes for high-throughput batch reactor metabolism experiments
Source: PLoS One. 2023 Jul 11;18(7):e0284256. doi: 10.1371/journal.pone.0284256 (PMC10335663; doi:10.1371/journal.pone.0284256)
Supplement: S19 File — Oxygen saturation values were calculated using the equation DOsalt = DO−qS where DOsalt is the dissolved oxygen concentration (in mg/L) of air-saturated salt water, and DO is the dissolved oxygen concentration (mg/L) of air-saturated distilled water, and q is approximated using the equation: q = −0.1903t+12.892, where t is temperature in degrees Celsius. (DOCX) [file pone.0284256.s019.docx]

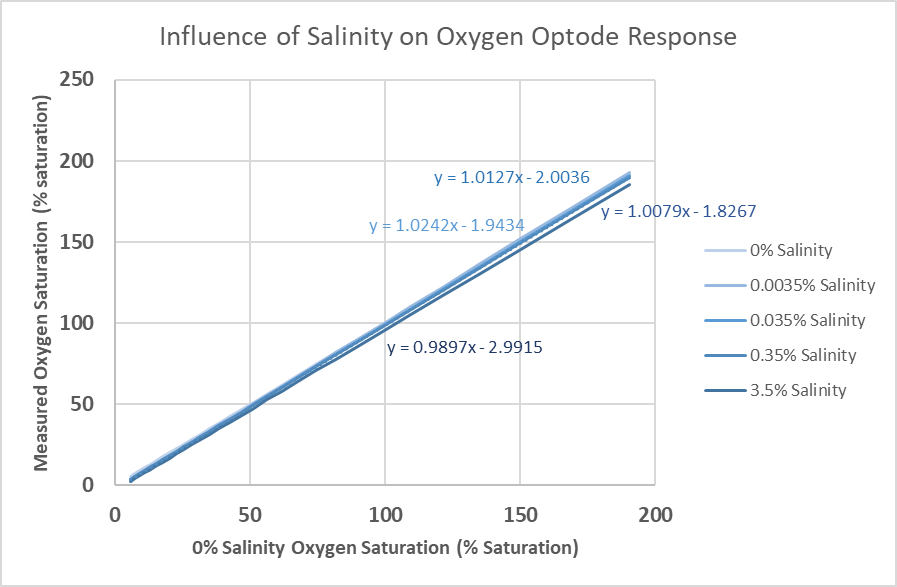


S19: Salinity values were varied by adding NaCl to the reservoir at concentrations ranging from 0% to 3.5% (m/v). Oxygen saturation values were calculated using the equation ${DO}_{salt}=DO-qS$ where DO_salt_ is the dissolved oxygen concentration (in mg/L) of air-saturated salt water, and DO is the dissolved oxygen concentration (mg/L) of air-saturated distilled water, and q is approximated using the equation: $q= -0.1903t+12.892$, where t is temperature in degrees Celsius.
