# Supplementary material for: Metabolic multireactor: Practical considerations for using simple oxygen sensing optodes for high-throughput batch reactor metabolism experiments
Source: PLoS One. 2023 Jul 11;18(7):e0284256. doi: 10.1371/journal.pone.0284256 (PMC10335663; doi:10.1371/journal.pone.0284256)
Supplement: S20 File — The values in the plot above have been adjusted to account for differences in temperature between vials. Overall, pH was not observed to significantly influence optode response (outside the ±3% range) except at pH below ~4.5. (DOCX) [file pone.0284256.s020.docx]

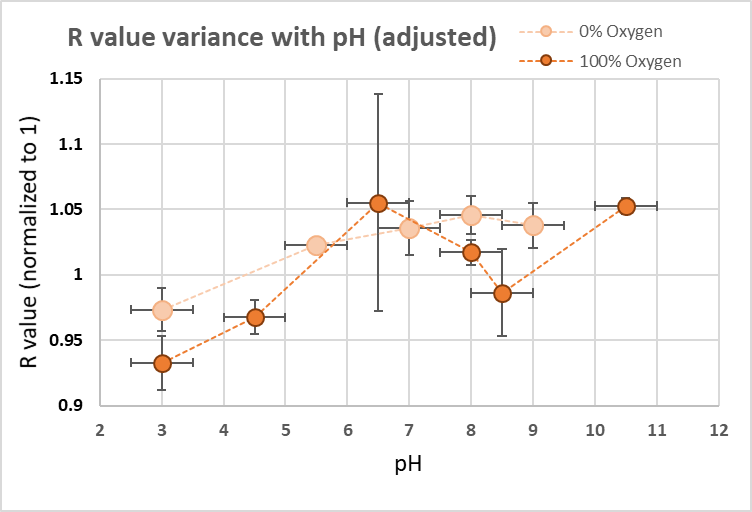


S20: pH values were increased and decreased by adding sodium hydroxide and hydrochloric acid, respectively, to vials containing varied concentrations of oxygen. The values in the plot above have been adjusted to account for differences in temperature between vials. Overall, pH was not observed to significantly influence optode response (outside the ±3% range) except at pH below ~4.5.
