# Supplementary material for: Metabolic multireactor: Practical considerations for using simple oxygen sensing optodes for high-throughput batch reactor metabolism experiments
Source: PLoS One. 2023 Jul 11;18(7):e0284256. doi: 10.1371/journal.pone.0284256 (PMC10335663; doi:10.1371/journal.pone.0284256)
Supplement: S21 File — No significant deviation from results obtained at 0mg/L nitrate were observed. (DOCX) [file pone.0284256.s021.docx]

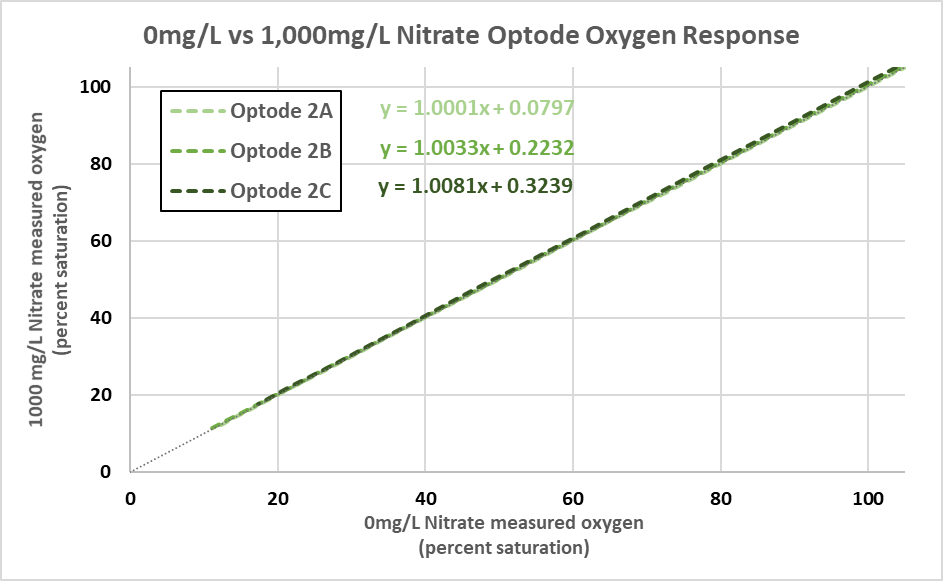


S21: Optode response in the presence of nitrate was tested by amending the reservoir solution with sodium nitrate at a concentration of 1,000mg/L. No significant deviation from results obtained at 0mg/L nitrate were observed.
