# Supplementary material for: Metabolic multireactor: Practical considerations for using simple oxygen sensing optodes for high-throughput batch reactor metabolism experiments
Source: PLoS One. 2023 Jul 11;18(7):e0284256. doi: 10.1371/journal.pone.0284256 (PMC10335663; doi:10.1371/journal.pone.0284256)
Supplement: Attachment — Submitted filename: review_Kaufmann.pdf [file pone.0284256.s022.pdf]

The manuscript entitled "Metabolic Multireactor: Practical Considerations for Using Simple Oxygen Sensing Optodes for High-throughput Batch Reactor Metabolism Experiments," authored by Kaufman and colleagues, introduces a novel incubation setup that enables simultaneous measurement of oxygen consumption in multiple reactors. The authors conducted meticulous testing of the system and provided comprehensive supplementary information, including all CAD designs. Overall, the study presents a valuable contribution to the field and showcases the potential of this methodology for high-throughput batch reactor metabolism experiments.

The proposed system has the potential for wide applicability in various fields of research, including marine, aquatic, and terrestrial studies. This broad scope of potential applications makes the manuscript an appealing read for a broad readership, and its suitability for publication in PLOS ONE is evident. As the reviewer, I have opted for a "major revision" decision, as I believe there is room for improvement in certain sections of the manuscript. Including these clarifications will improve the overall clarity and understanding of the experimental design.

General comments

1. **Heterogeneity measurements**: In the introduction, the authors discuss the importance of heterogeneity measurements, which I completely agree with. In my opinion, the proposed system has an key advantage that is currently not addressed: it can directly resolve heterogeneity on the micrometer to millimeter scales. By imaging the roughly 2 cm dye spots, the authors can extract information about the variability of the sensor-dye. This approach would only require determination of the minimum resolvable spatial resolution.
2. **Improve precision through higher temporal resolution**: The manuscript does not explore the potential benefits of increasing the temporal resolution of the readouts to enhance the accuracy of rate measurements. This is an interesting avenue for future research that could be discussed or acknowledged within the manuscript. By improving the temporal resolution of the system, researchers may be able to obtain more precise measurements of metabolic rates (for comparison doi.org/10.1371/journal.pone.0089369).
3. **IR blocking:** For the applied sensor-dye (PtTFPP) the IR blocking is not really important, as most of the emission is occurring in the lower red range (600 nm – 680 nm). This might however change for other dyes that are utilized by the community. This should be clarified and the sections on the IR blocking should be toned down.
4. **Terminology**: Throughout the manuscript, the author uses terms such as "metabolism of organic material," "microbial metabolism", "aerobic metabolism", "microbial respiration", and "consumption" interchangeably, despite the fact that these terms describe different processes. While the measurements obtained are primarily oxygen consumption measurements, which integrate a range of processes, including microbial respiration and oxidation of reduced material. It is important to clearly describe this link between organic carbon remineralization and oxygen respiration in the introduction and use consistent terminology throughout the manuscript. I recommend to use "oxygen consumption," to accurately reflect the measurements obtained and avoid confusion.
5. **Calibration**: The authors are performing and describing in detail the calibration procedure, however the Stern-Volmer equations are missing and should be integrated in the method

section then it also becomes clearer to which parameter the authors are referring in **table 1**. In my opinion the limit of detection (1.1%) is very conservative and it does not reflect the actual data (Figure 9, Zoom into 0-0.6 mg/L and add the 1.1% line).

6. **Literature**: The reference list is relatively short and does not reflect the breadth of research in this field. The proposed system has the potential to be applied in various environments, including marine, aquatic, and terrestrial studies. Therefore, it would be beneficial for the authors to expand their literature review and include more relevant studies. Additionally, some recent novel developments have addressed heterogeneity on the micrometer scale, which should also be considered in the manuscript.: doi.org/10.1016/j.crmeth.2022.100216

Line and figure specific comments

Figures: To reduce the total number of figures in the manuscript, I recommend combining multiple panels into a single figure. Rather than presenting each panel as a separate figure, combining them will simplify the presentation and make it easier for readers to follow the results. This approach will also reduce the total number of figures, making the manuscript more concise and easier to navigate. Figure 1 and figure 2 can be combined. Add scale bar to figure 1 (panel bottom left).

Abstract: The abstract could be restructured for better clarity. It may be more effective to begin with the importance/introduction and limitations of current systems, followed by the setup and advantages of the proposed system. Also, it may not be necessary to include the reference to Larsen et al.. Instead, it could be stated that ratio-metric sensor-dyes are used, along with the names of the specific dyes.

Line 32: See general comment 4

Line 46: The authors should give examples of such “whole-system approaches”

Line 52: The authors should describe what is meant with “manipulative experiments”

Line 62: The reference [11] seems to not reflect the statements made.

Line 69-70: I disagree to the statement that optodes are traditionally “used to obtain two-dimensional data”. Traditionally optodes are used in the context of microsensors (doi.org/10.1016/S0925-4005(97)80168-2)

Line 118: Effects of bleaching can also be reduced, calibration is improved, etc. pp. Further, while I agree that it is not necessary to provide an in-depth description of the dyes and their chemical properties, it is important to mention which reference dye was used in the manuscript. Additionally, the distinction between the antenna and reference dyes could be clarified for readers. It is the same dye, but the reference dye also serves as an antenna dye, facilitating energy transfer. Also write out once for what PtTFPP actually stands for.

Line 274: It is rather the “variability” then the “variance” that is testes

Line 337: The wording (“Lifetime test”) is a bit confusing as the lifetime of the quenching can also be used for calibration.

Line 465: The authors are very conservative with their statements regarding how inexpensive their system is. Commercial solutions for similar 4-channel modules are substantially more expensive, such as

the Pyroscience system, which costs approximately $10,000 and that does not include any of the spots and additional lab equipment (and allows only the read-out of 4 spots). It may be worth making stronger statements regarding the cost-effectiveness of the proposed system in comparison to existing commercial solutions.
